# Serum and thyroid tissue level of let-7b and their correlation with TRAb in Graves’ disease

**DOI:** 10.1186/s12967-018-1565-9

**Published:** 2018-07-05

**Authors:** Xinxin Chen, Fengjiao Huang, Yicheng Qi, Mengxi Zhou, Qinglei Yin, Ying Peng, Yulin Zhou, Guang Ning, Shu Wang

**Affiliations:** Department of Endocrinology, Shanghai Clinical Center for Endocrine and Metabolic Diseases, Shanghai Institute of Endocrine and Metabolic Diseases, Ruijin Hospital, Shanghai Jiao Tong University Medical School, 197 Ruijin 2nd Road, Shanghai, 200025 People’s Republic of China

**Keywords:** Circulating miRNAs, Let-7b, Graves’ disease, Thyrotropin receptor antibody

## Abstract

**Background:**

Abnormal microRNAs (miRNAs) were reported to be involved in the mechanism of Graves’ disease (GD). Dysregulated miRNAs may be overlapping in different cells and can be secreted to circulation. We chose miRNAs which were previously reported to be differentially expressed in peripheral blood mononuclear cells (PBMCs) in patients with GD with different disease stage, detected the expression of those miRNAs in serum, corroborated the findings in thyroid tissue, and validated the target gene in vitro to investigate the possible role of circulating miRNAs in GD.

**Methods:**

A total of 54 individuals with untreated GD, 12 individuals with GD in remission and 14 disease-free controls were enrolled. The expression of miR-142-3p, miR-154-3p, miR-431-3p, miR-590-5p, and let-7b was detected in the serum. Ten thyroid tissue samples from patients with GD and six disease-free thyroid samples were used for further validation. The potential target genes were identified and validated in vitro.

**Results:**

miR-142-3p, miR-154-3p, miR-431-3p, miR-590-5p, and let-7b were present in serum and two of them (miR-142-3p and let-7b) were significantly increased in serum of patients with untreated GD (for serum miR-142-3p, *P* = 0.033, for serum let-7b, *P* = 0.026) and gradually decreased to normal levels in patients with GD in remission. Correlation analysis showed that let-7b level was strongly correlated with TRAb level (*r* = 0.305, *P* = 0.001). let-7b directly inhibited promyelocytic leukemia zinc finger (PLZF) expression and increased the expression of TSHR in thyroid cells in vitro. Furthermore, let-7b levels in GD thyroid tissue were found to be inversely correlated with PLZF levels (*r *= − 0.849, *P* = 0.033). Decreased PLZF and increased TSHR was validated in thyroid tissue in patients with GD.

**Conclusions:**

The present study confirmed that a portion of miRNAs in PBMCs were also presented and differentially expressed in serum and thyroid tissue. Upregulated in all these three compartments, let-7b may be used as a disease biomarker and therapeutic targets in patients with GD. Circulating let-7b had a strong correlation with disease severity and let-7b may participate in the production of TRAb via targeting PLZF in patients with GD.

**Electronic supplementary material:**

The online version of this article (10.1186/s12967-018-1565-9) contains supplementary material, which is available to authorized users.

## Background

Graves’ disease (GD) is the most common cause of hyperthyroidism in the clinic, affecting about 1% of the general population [1, 2]. GD is an antibody-mediated organ-specific autoimmune disease and is initiated by the interplay of susceptibility genes, environment, and immune disorders [3, 4]. It is well established that human thyrotropin receptor (hTSHR) is the primary autoantigen of Graves’ disease. The circulating autoantibodies to TSHR are called thyrotropin receptor antibodies (TRAb) which can mimic the role of TSH, binding to TSHR and leading to overproduction of thyroid hormones and thyroid hyperplasia [5]. TRAb is detectable in 95–100% untreated GD patients and decreased by treatment and, when they persist, may predict recurrence [6]. Thus, TRAb is disease specific and plays a central role in the etiology of GD. However, the mechanism of the production of TRAb remained unclear.

MicroRNAs (miRNAs) are noncoding RNAs that interact with their target RNAs in a sequence-dependent manner [7]. miRNAs are involved in several important biological processes including immunity, apoptosis, cell differentiation and development, proliferation, and metabolism [8]. Similar to other autoimmune diseases, recently studies concentrated on the specific miRNAs profiles in GD. The importance of miRNAs in the pathogenesis of GD was highlighted in many reports [9–12]. Originating from the active or passive release by cells, circulating miRNAs were an emerging area in recent years. Evidence suggested that circulating miRNAs can be used as biological markers for diseases such as tumors and inflammatory diseases [13, 14]. Studies demonstrated that circulating miRNAs had a strong correlation with disease severity in patients with GD. Yamada et al. explored circulating miRNA expression profiles in 17 patients with GD through microarray. miR-16, miR-22, miR-375, and miR-451 were significantly increased in circulation in patients with GD [15]. Increased circulating miR-23b-5p and miR-92a-39, as well as decreased circulating let-7g-3p and miR-339-5p were associated with intractable GD [16]. Interestingly, circulating miRNAs were reported to be disseminated through the extracellular fluid to reach target cells and participated in the intercellular communication. Although the specific mechanism remained unknown, miRNA can be transferred between different cells through apoptotic body or extracellular vesicles were observed in vitro systems. Circulating miRNAs might have hormone-like effects leading to widespread responses within different types of cells [17]. Thus, circulating miRNAs might dysregulated in different compartments and paly crucial roles in the pathogenesis of the disease. In multiple sclerosis, miR-21, miR-142-3p, miR-146a, miR-155 and miR-326 was found to be upregulated in both PBMCs and brain white matter lesions from patients and mouse model as well [18]. Marazuela et al. investigated the differential expression profiles of miRNAs in thyroid tissue in GD samples, and then detected these miRNAs in serum. The results showed that some miRNAs that were highly expressed in thyroid tissues were also highly expressed in the serum and serum let-7d was strongly correlated with disease severity [19].

The differential expression of miRNAs in peripheral blood mononuclear cells (PBMCs) in untreated GD patients was reported in our previous study, where miR-142-3p, miR-154-3p, miR-431-3p, miR-590-5p, and let-7b were significantly differential expressed [10]. Those miRNAs have been reported in several autoimmune diseases such as rheumatoid arthritis [20], systemic lupus erythematosus [21], multiple sclerosis [18, 22] and psoriasis [23] (shown in Additional file 1: Table S1). We hypothesized that those miRNAs, which were involved in various inflammatory signaling pathways, may be presented and differentially expressed in serum and tissue and may participate in the pathogenesis mechanism of GD and the production of TRAb. However, very few studies focused on the expression of those miRNAs candidates in serum or thyroid tissue of patients with GD. Therefore, we detected the expression of these miRNAs in serum in GD patients with different stage, correlated their expression with GD patients’ clinical data, identified the target genes and validated in thyroid tissue to investigate the possible pathogenesis role of circulating miRNAs in patients with GD.

## Methods

### Enrollment of patients and disease-free controls

Patients and disease-free controls were enrolled from the outpatient department of Endocrinology of Ruijin Hospital affiliated to Shanghai JiaoTong University Medicine School. The diagnosis of GD was based on clinical manifestation and laboratory examination. Typical manifestations included heat intolerance, fatigue, increased appetite, increased sweating, weight loss, muscle weakness, tremors, and diffusely enlarged thyroid gland. Laboratory examinations included increased serum concentration of free triiodothyronine (FT3), free thyroxine (FT4), decreased serum concentration of sensitive thyrotropin (sTSH), and high tilters of TRAb. Patients with untreated GD were all newly diagnosed and untreated in our study. Patients were considered to be in remission when thyroid function returned to normal (TSH, FT3, and FT4 within the reference range and negative TRAb) under the treatment of anti-thyroid drugs. The diagnosis of Graves’ disease was confirmed by the experienced physician or endocrinologist in Ruijin Hospital affiliated to Shanghai JiaoTong University. Further, a group of subjects without thyroid disease as disease-free controls were also recruited. Disease-free controls were subjects who had no personal and family history of thyroid disease, with normal thyroid ultrasound imaging, and serum TSH, FT3/FT4 within reference range and negative thyroid antibody.

### Serum microRNA extraction

Three to 5 mL peripheral blood were collected and centrifuged at 1900*g* (3000 rpm) for 10 min at 4 °C to isolate serum. Serum isolation was completed within 4 h after collection. miRNA was isolated from 200 μL of serum using the miRNeasy Serum/Plasma Kit (Qiagen) following the manufacturer’s protocol. miRNeasy Serum/Plasma Spike-In Control (*C. elegans miR*-*39*) was added to serum as a control before miRNA extraction.

### Thyroid tissue miRNA extraction

We collected 10 thyroid tissue samples from patients with GD and six disease-free thyroid tissue samples confirmed by pathological examination from thyroid nodule patients who had undergone thyroidectomy between April 2017 and October 2017 at Ruijin Hospital. miRNA extraction from thyroid tissue samples was performed using an miRNeasy Mini Kit (Qiagen) by following the manufacturer’s protocol.

### Real-time reverse transcription polymerase chain reaction

cDNAs were synthesized using a miScript Reverse Transcription Kit (Qiagen, Hilden, Germany). The expression levels of miRNAs were confirmed with a miScript SYBR Green PCR kit and miRNA-specific primers (Qiagen). Commercially available primers purchased from Qiagen were used as follows: Hs_miR-142-3p miScript Primer Assay, Hs_miR-154-3p miScript Primer Assay, Hs_ miR-431-3p miScript Primer Assay, Hs_miR-590-5p miScript Primer Assay, and Hs_let-7b miScript Primer Assay. *RNU6*-*2* served as the internal normalization control for the expression in thyroid tissue. *C. elegans mir*-*39* served as the internal normalization control for the expression in serum. Light Cycler 480 software (Roche Applied Science, Indianapolis, IN, USA) was used to analyze data. All reactions were performed in triplicate and the results were calculated by determining the ΔΔCt value.

### Cell isolation and cell culture

Primary thyroid cells were isolated from fresh thyroid tissue from normal thyroid tissue. Primary thyroid cells and Nthy-ori3-1 cells were maintained in RMPI 1640 supplemented with 10% FBS and 1% penicillin–streptomycin at 37 °C in 5% CO_2_. 293T cells were maintained in DMEM supplemented with 10% FBS and 1% penicillin–streptomycin at 37 °C in 5% CO_2_. The cells were passaged every 3 days.

### The transient transfection

Lipofectamine 3000 (Invitrogen, Carlsbad, CA, USA) was used as a transfection reagent. let-7b mimics, let-7b inhibitors, and mimic control or negative control were mixed with the transfection reagent and then added to the cells, followed by incubation for 24 h at 37 °C in 5% CO_2_.

### Western blot analysis

Cell lysates were subjected to western blot analysis according to standard protocols. After blocking, the membranes were incubated overnight at 4 °C with primary antibodies to PLZF (1:1000; SAB, USA) or TSHR (1:1000; Proteintech, USA). GAPDH (1:3000; Cell Signaling Technology, USA) was used as a normalization control. Next, the membranes were incubated with the appropriate horseradish peroxidase-conjugated secondary antibody (1:2000; Cell Signaling Technology, USA). Protein bands were illuminated using ECL Prime Western Blotting Detection Reagent (GE Healthcare, Little Chalfont, Buckinghamshire, UK).

### Luciferase activity assay

The pmiR-RB-REPORT vector (RIBOBIO) was used to clone the PLZF 3′UTR sequence containing the putative miR-4443 binding sites, designated as wild type 1 (binding sites: 1889–1895; PLZF-WT1) and wild type 2 (binding sites: 4766–4773; PLZF-WT2). Reporter plasmids of the corresponding mutation (Mut1 and Mut2) were also constructed. The PLZF-WT1 (1889–1895), PLZF-WT2 (4766–4773), and mutant type (PLZF-mut1 and PLZF mut2) constructs were amplified and cloned downstream of a luciferase reporter gene in the pmiR-RB-REPORT vector. HEK293T cells were seeded in 24-well plates the day before transfection. For each well, 100 ng of wild-type or mutant reporter plasmid was transiently co-transfected with miRNA mimics or negative control using Lipofectamine 3000 (Invitrogen). Cell lysates were harvested 48 h after transfection, and the cells were subjected to a Dual-Luciferase Reporter Assay System (Promega) according to the manufacturer’s instructions. Renilla luciferase activity was normalized to firefly luciferase activity to control for transfection efficiency.

### Statistical analysis

SPSS version 19.0 were used for statistical calculations. All figures were completed with Graph Pad Prism 6.0. Data are presented as mean ± SD. *P *< 0.05 was considered significant. Statistical difference was evaluated using Mann–Whitney U-test for continuous variables among three groups. The Chi square test was used to test for categorical variables. Correlations between the different variables were analyzed by Spearman correlation.

## Results

### The general clinical characteristics of the study population

A total of 54 patients with untreated GD, 12 patients with GD in remission, and 14 disease-free controls were enrolled in our study. The general characteristics of patients and disease-free controls are summarized in Table 1. There was no significance in age and gender composition between untreated GD patients and disease-free controls. FT3, FT4, TRAb, TPOAb, and TgAb were highest and TSH was lowest in patients with untreated GD. FT3, FT4, TSH and TRAb were returned to normal in patients with GD in remission.

**Table 1:** Clinical characteristics of the study population

	Disease-free controls	Untreated GD	GD in remission
N (F/M)	14 (9/5)	54 (38/16)	12 (9/3)
Age (years)	31.57±6.81	38.87±11.30	47.55±11.61*
FT3 (pmol/L)	4.62±0.51^#^	26.90±14.17*	4.13±0.49^#^
FT4 (pmol/L)	13.76±1.57^#^	41.44±28.87*	13.47±1.10^#^
TSH (mIU/L)	1.93±1.17^#^	0.32±2.33*	2.38±0.95^#^
TRAb (IU/L)	0.46±0.24^#^	17.15±12.88*	0.75±0.13^#^
TPOAb (IU/mL)	19.02±68.15^#^	435.77±366.92*	274.38±157.71
TgAb (IU/mL)	6.01±13.57^#^	209.71±275.97*	74.25±61.31

* Compared to disease-free controls, *P *< 0.001

^#^ Compared to untreated GD, *P *< 0.001

### The expression of miR-142-3p, miR-154-3p, miR-431-3p, miR-590-5p, and let-7b in serum and correlation with TRAb in Graves’ disease

**Fig. 1:**
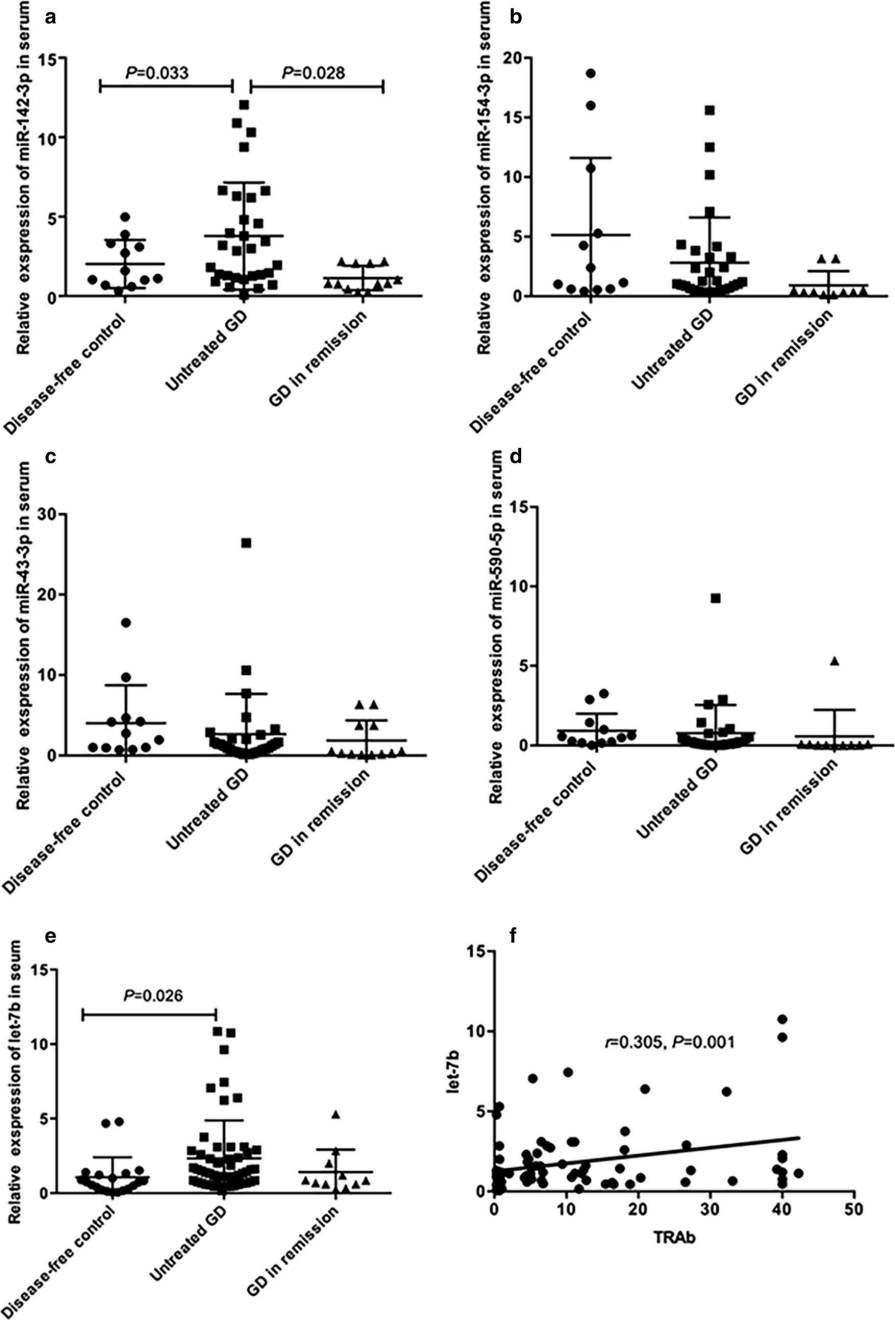
Relative expression of miR-142-3p, miR-154, miR-431-3p, miR-590-5p and let-7b in serum and correlation with TRAb. Relative expression of miR-142-3p (**a**), miR-154 (**b**), miR-431-3p (**c**), miR-590-5p (**d**) and let-7b (**e**) expression in patients with hyperthyroidism with untreated Graves’ disease (GD), patients in remission with TRAb-negative GD, and disease-free controls. **f** The correlation between the expression levels of let-7b in serum and TRAb levels

The expression of miR-142-3p, miR-154-3p, miR-431-3p, miR-590-5p and let-7b were detected in serum. Compared to disease-free controls, serum miR-142-3p and let-7b expression was significantly increased in patients with untreated GD (for serum miR-142-3p, *P* = 0.033; for serum let-7b, *P* = 0.026), and gradually decreased to normal under the anti-thyroid treatment in patients with GD in remission (Fig. 1a, e). There was no significance difference in serum miR-154-3p, miR-431-3p, and miR-590-5p levels among the three groups (Fig. 1b–d). Correlation analysis showed that only serum let-7b level was positively correlated with TRAb level (*r* = 0.305, *P* = 0.001, Fig. 1f). Serum miR-142-3p level was not correlated with TRAb (*r* = 0.231, *P* = 0.147).

### PLZF is a direct target gene of let-7b

**Fig. 2:**
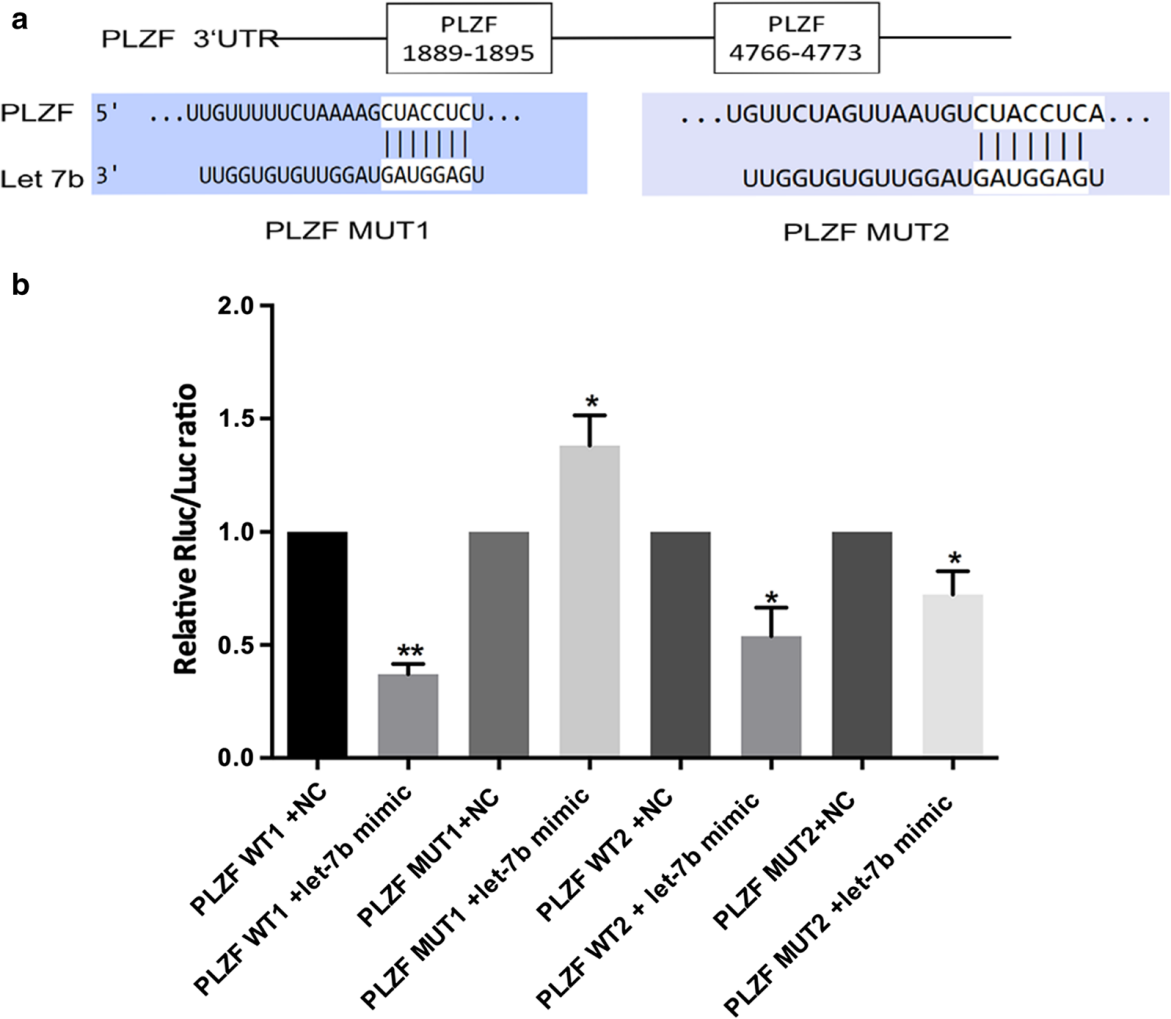
PLZF is a direct target gene of let-7b. **a** 2 potential binding sites between let-7b and PLZF. 293T cells were co-transfected with the PLZF gene sequence with wild-type and mutated let-7b. Fluorescence intensity was measured 24 h later using the Dual-Luciferase Reporter Assay System and the results are shown in (**b**)

It is well known that miRNAs function by regulating their target genes. In the present study, the putative let-7b target genes were predicted using miRbase and TargetScan. PLZF possesses two potential complementary sites for the let-7b seed region in its 3′UTR (Fig. 2a). To further explore the direct binding and repression effect between let-7b and PLZF, a luciferase activity assay was performed. Co-transfection of let-7b mimics and PLZF-WT1 in HEK293T cells markedly reduced luciferase activity compared with activity in the negative control (*P* < 0.001, Fig. 2b). However, co-transfection of let-7b mimics and PLZF-mut1 in HEK293T cells did not reduce luciferase activity. Additionally, no difference in luciferase activity was observed between PLZF-WT2 and PLZF-mut2. The luciferase reporter assay showed that let-7b binds more at the putative binding sites of 1889–1895.

### Expression of let-7b, PLZF, and TSHR in thyroid tissue

**Fig. 3:**
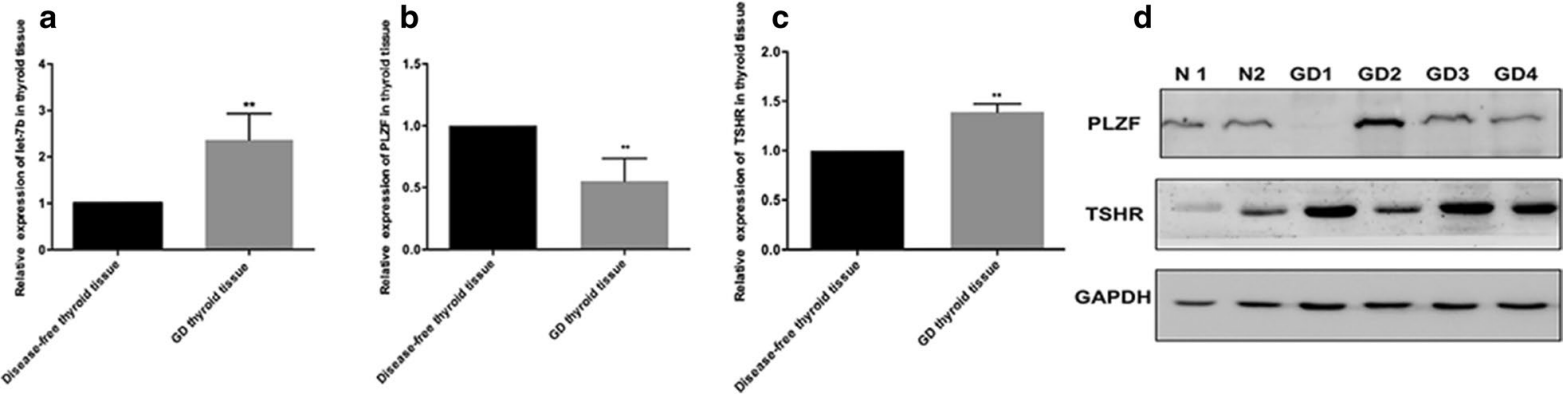
Expression of let-7b, PLZF, and TSHR in thyroid tissue. Relative expression of let-7b, PLZF, and TSHR in thyroid tissue. The expression of *let*-*7b* (**a**), *PLZF* mRNA (**b**), and *TSHR* mRNA (**c**) in thyroid tissues from individuals with Graves’ disease (GD) and normal thyroid tissues by real-time PCR. **d** Protein levels of PLZF and TSHR in GD thyroid tissue. **P < 0.001

let-7b level was strongly correlated with TRAb level, and PLZF, which inhibits TSHR expression in the thyroid, was its target gene. Thus, we validated the expression of let-7b, PLZF, and TSHR in thyroid tissue. Compared to levels in normal thyroid tissue from patients with thyroid nodules, the expression of let-7b was significantly increased in thyroid tissue in patients with GD (*P *< 0.001, Fig. 3a). PLZF expression significantly decreased while TSHR level significantly increased in GD thyroid tissue as determined by Real-time PCR and western blotting (Fig. 3b–d). Additionally, correlation analysis showed that the expression of let-7b was significantly inversely correlated with PLZF level in thyroid tissue (*r *= − 0.849, *P* = 0.033).

### Let-7b increases TSHR expression in thyroid tissue via inhibiting PLZF

**Fig. 4:**
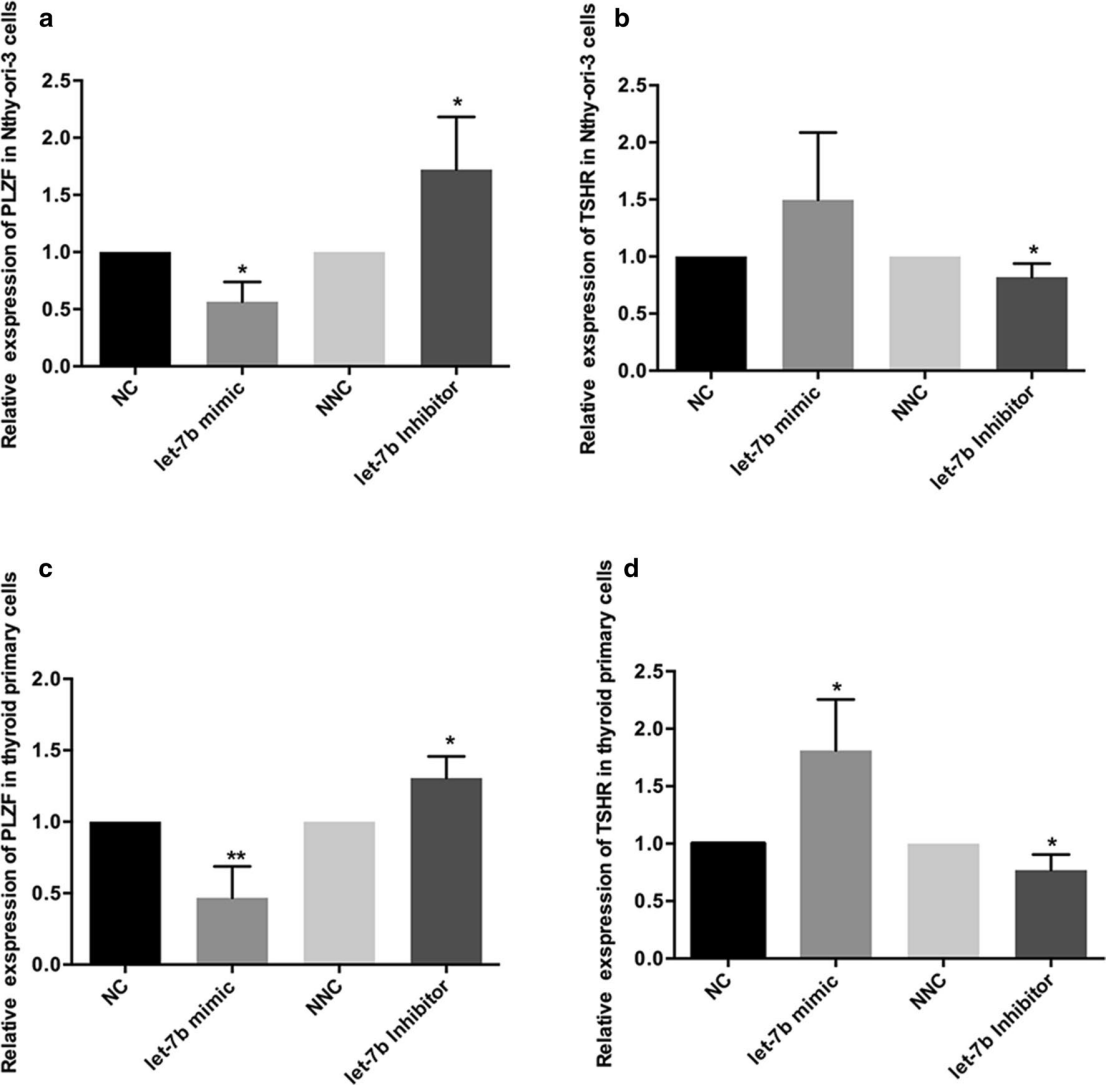
let-7b regulates TSHR expression via targeting PLZF. The expression of PLZF and TSHR in thyroid tissue. Thyroid primary cells were isolated from fresh thyroid tissue and cultured in vitro. Negative control or let-7b mimics, Inhibitor negative control, or let-7b inhibitors were transfected into primary thyroid cells (**a**, **c**) and human normal thyroid primary cells (**b**, **d**) and *PLZF* mRNA (**a**, **b**) and *TSHR* mRNA (**c**, **d**) was detected by real-time PCR. Results were calculated by three independent experiments, *P < 0.05, **P < 0.001

To explore whether let-7b increased the expression of TSHR via inhibiting PLZF in thyroid cells, Nthy-ori3-1 and primary thyroid cells were transfected with let-7b mimics or let-7b inhibitors in vitro. After transfection with let-7b mimics, reduced *PLZF* mRNA and increased *TSHR* mRNA levels were found in both Nthy-ori3-1 and primary thyroid cells, whereas let-7b inhibition resulted in increased *PLZF* mRNA and decreased *TSHR* mRNA expression (Fig. 4).

## Discussion

Circulating miRNAs are emerging area in autoimmune disease and attracted our attention. Because of their highly stable and reproducible characteristics, circulating miRNAs are reliable biological markers for diseases such as tumors and inflammatory diseases [13, 14]. Overexpression of circulating miR-191-5p, miR-24-3p, miR-128-3p, and miR-376c-3 was detected in patients with multiple sclerosis [24]. In addition, miR-24-3p and miR-128-3p tended to associate with dysfunction accumulation and disease activity, respectively [25]. Limited research reported the circulating miRNAs in patients with GD. Yamada et al. [15] found that miR-16, miR-22, miR-375, and miR-451 were significantly increased in circulation in patients with GD. Hiratsuka et al. demonstrated that let-7g and miR-339-5p levels were decreased in patients with GD in remission compared with levels in patients with intractable GD [16].

It was thought circulating miRNAs have hormone-like effects leading to widespread responses within different types of cells [17]. Circulating miRNAs were originated from the passive released from cells and could reach to remote target cells to communicate. Differential expression of miRNAs in specific cells might lead to differential expression in circulation. And, differential expression of miRNAs may be overlapping in different cells. In multiple sclerosis, miR-21, miR-142-3p, miR-146a, miR-155 and miR-326 were found to be upregulated in both PBMCs and brain white matter lesions from patients and mouse model [18]. Nagarkatti et al. demonstrated miR-155 could serve as diagnostic markers and therapeutic targets in multiple sclerosis since miR-155 was all upregulated in plasma, PBMCs and brain white matter lesions from patients with multiple sclerosis. Besides, evidence from animal studies suggested that suppression miR-155 could inhibit the development of Th1 and Th17 cells and anti-miR-155 could inhibit EAE development [26, 27]. Thus, some overlapping in the differentially expression miRNA profiles in disease may play crucial roles in disease and could be used as potential therapeutic target.

Based on our previous study, miR-142-3p, miR-154-3p, miR-431-3p, miR-590-5p, and let-7b were differentially expressed in PBMCs in untreated GD patients [10]. Those miRNAs were involved in various pathways such as NF-κB pathways, TNF pathways, Toll-like receptor pathways which were closely related with the pathogenesis of GD and were reported in previous literatures to be related with autoimmune diseases (shown in Additional file 1: Table S1) [28, 29]. In this regard, we investigated the expression of miR-142-3p, miR-154-3p, miR-431-3p, miR-590-5p, and let-7b in serum of patients with GD in this study. The results showed that serum miR-142-3p and serum let-7b levels were significantly increased in patients with untreated GD and recovered in GD patients with remission.

**Fig. 5:**
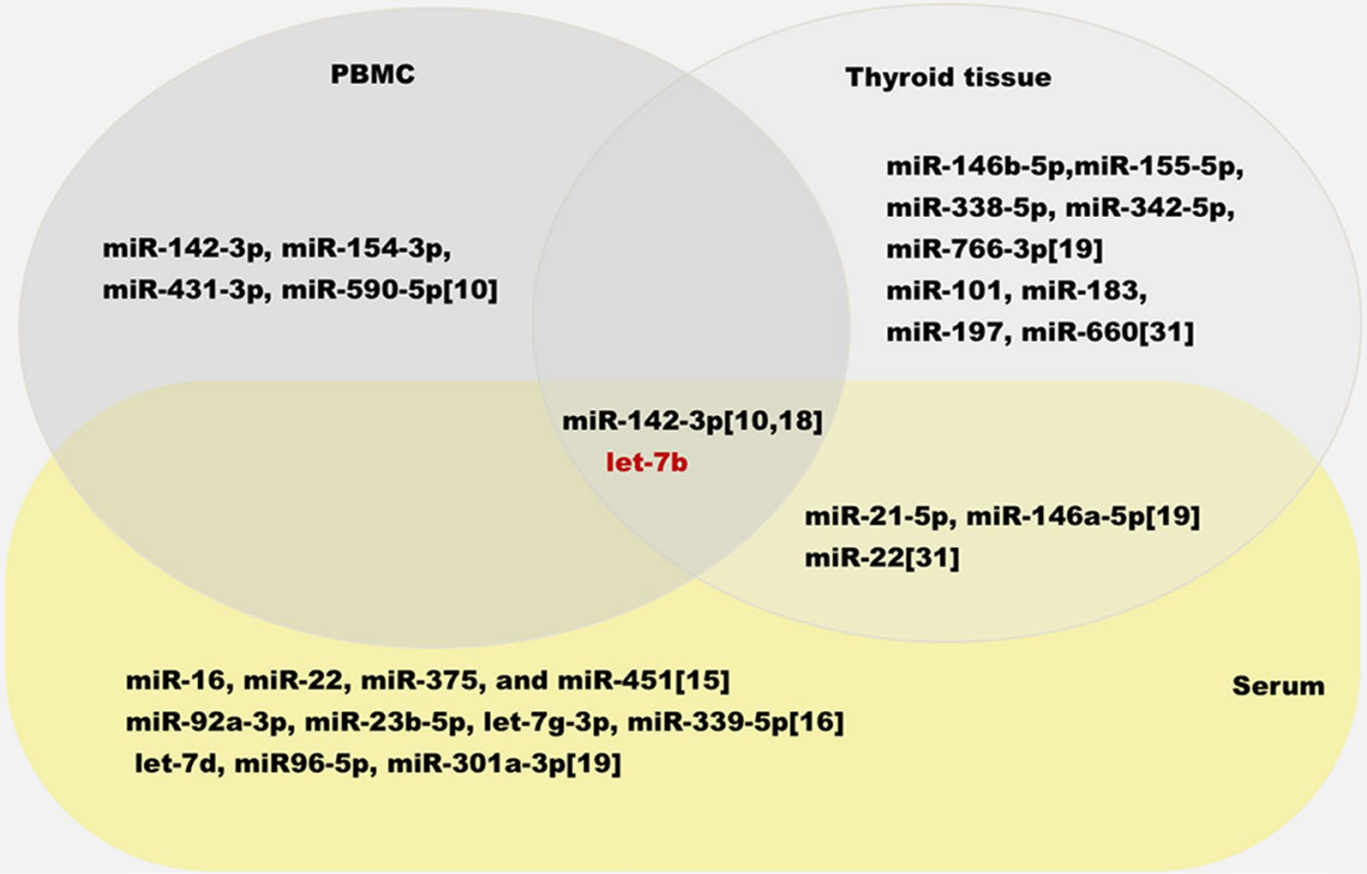
Overlapping between dysregulated miRNAs in serum and peripheral blood mononuclear cells and thyroid tissue. The data of dysregulated miRNAs was compiled from miRNAs expression profiling studies in serum [15, 16, 19], in peripheral blood mononuclear cells [10] and in thyroid tissue [19, 30]. Identical dysregulated miRNAs were shown in overlapping area. Non-identical dysregulated miRNAs were shown in single area

In addition, we summarized dysregulated profiles of miRNAs in patients with GD based on previous literatures (Fig. 5). Coupled with our results, we found some overlapping of these miRNAs in patients with GD. miR-22 was reported by Qin in lesioned tissue of GD and reported by Yamada et al. to be increased significantly in circulation in patients with GD [15, 30]. Marazuela et al. demonstrated that miR-142-3p, miR-21-5p, miR-146a-5p were dysregulated in both thyroid tissue and serum [19].

Consistent with the results of their study, miR-142-3p and let-7b level was significantly increased in serum in our study. Dysregulated in three compartments. miR-142-3p was upregulated in serum and thyroid tissue and downregulated in PBMCs in patients with GD. let-7b was upregulated in serum, thyroid tissue and PBMCs in patients with GD. miR-142-3p is abundant in cells of hematopoietic origin and has gained considerable attention for its role in regulating immune response [31]. Software predicted that miR-142-3p was involved in inflammtory pathways such as B cell receptor signaling pathway and TNF signaling pathway, which was responsible for the production of antibody. Especially, evidence suggested that miR-142-3p impaired the inhibitory Treg cells on the proliferative response and cytokine production of CD4+ CD25− T cells [32]. The impaired inhibitory function of Treg cells in patients with GD were confirmed in previous literatures [33, 34]. Thus, dysregulated miR-142-3p might exacerbated the immune process via impairing Treg cell function in patients with GD. However, there was no correlation between miR-142-3p and thyroid function parameters in our study, and we did not validate furtherly. Instead, we found that let-7b level strongly correlated with TRAb level in this study, which was used as an indicator of GD diagnosis and disease severity, suggesting a possible role of let-7b in the mechanism of GD and TRAb production.

let-7b belongs to the let-7 miRNA family, which is one of the important families of miRNAs. The members of let-7 miRNA family include let-7a-1, let-7a-2, let-7a-3, let-7b, let-7c, let-7d, let-7d, let-7e, let-7f-1, let-7f-2, let-7g, let-7i, mir-98, and mir-202 [35]. Circulating let-7 was reported in various other autoimmune diseases. let-7b levels were increased in the serum of patients with immunoglobulin A nephropathy and were a reliable predictor of the probability of having immunoglobulin A nephropathy [36]. Gandhi et al. [37] identified circulating miRNAs (let-7 and miR-92) that were differentially expressed in relapsing-remitting MS patients compared to secondary progressive MS patients. Mature let-7 miRNAs are highly conserved among different species and regulate multiple target genes affecting cell cycle and cell proliferation and differentiation [35, 38]. However, few reports have investigated circulating let-7b and its target gene in GD.

Promyelocytic leukemia zinc finger (PLZF), the target gene of let-7b, was validated in this study. There are two putative binding sites in PLZF (positions 1889–1895 and positions 4766–4773 in the 3′UTR of PLZF). As determined by a dual-luciferase reporter assay, let-7b mainly targeted the first binding site (positions 1889–1895 in the 3′UTR of PLZF). One recent study, whose results were consistent with ours, showed that let-7 miRNAs target the 3′UTR of *zbtb* mRNA to inhibit the expression of PLZF protein, which is required for innate-lineage T cell generation in the thymus. During the development of natural killer T (NKT) cells, the expression of let-7b is dynamically upregulated, directing NKT cells to eventually differentiate into IFN-producing NKT1 cells rather than IL-4-producing NKT2 cells or NKT17 cells that produce IL-17 [39]. PLZF is a transcription factor belonging to the BTB/POZ family, and functions by recruiting multiprotein complexes to regulatory gene elements [40, 41]. Findings of Stefan et al. suggest that PLZF can act as a transcriptional repressor of TSHR and downregulate TSHR expression [42]. TSHR is the primary autoantigen of GD. An enhanced presentation of TSHR on thyrocytes was reported in patients with GD [43], but the mechanism remains to be elucidated. We found upregulation of let-7b reduced the expression of PLZF and increased the TSHR expression in both primary normal thyroid cells and normal human thyroid cell lines. The opposite effect was observed when let-7b was downregulated in primary normal thyroid cells and normal human thyroid cell lines. We corroborated the expression of PLZF in thyroid tissue in patients with GD and found that PLZF was significantly decreased and inversely correlated with let-7b expression in the thyroid. These results suggested that changes in let-7b levels affect the expression of TSHR via PLZF. Therefore, we demonstrated that let-7b was involved in the production of TRAb by targeting PLZF.

The strengthen of our study was that we detected some miRNAs in serum and thyroid tissue in patients with GD. For the first time, we found that miR-142-3p and let-7b were dysregulated in three compartments. miR-142-3p was significantly upregulated in serum in patients with GD and could be used in the discrimination of GD patients from healthy control. let-7b was all upregulated in serum, thyroid tissue and PBMCs in patients with GD. let-7b significantly correlated with TRAb level in GD and let-7b may participate in the production of TRAb via PLZF. Therefore, let-7b may be a potential therapeutic target for patients with GD. One major limitation of this study was its relatively small sample size. Further studies need to confirm the utility of let-7b in patients with GD, which could help us further understand the pathogenesis of circulating miRNAs in GD and contribute to the development of potential therapeutic strategies in patients with GD.

## Conclusions

In the present study, we detected the expression of miRNAs in serum of patients with GD which was the differentially expressed in PBMCs. For the first time, we demonstrated that let-7b was upregulated in PBMCs, serum and thyroid tissue of patients with GD. Serum let-7b significantly correlated with TRAb level in GD and let-7b may participate in the production of TRAb via PLZF.

## Additional file

**Additional file 1: Table S1.** The predicted candidate gene, pathway and related other inflammatory disease of the selected microRNA candidates.

## Abbreviations

GD: Graves’ disease
TSHR: thyrotropin receptor
TRAb: thyrotropin receptor antibody
miRNAs: microRNA
PBMCs: peripheral blood mononuclear cells
TNF: tumor necrosis factor
TLRs: Toll-like receptors
PLZF: promyelocytic leukemia zinc finger
