# Supplementary material for: Serum and thyroid tissue level of let-7b and their correlation with TRAb in Graves’ disease
Source: J Transl Med. 2018 Jul 5;16:188. doi: 10.1186/s12967-018-1565-9 (PMC6034229; doi:10.1186/s12967-018-1565-9)
Supplement: Supplementary file 1 — Additional file 1: Table S1. The predicted candidate gene, pathway and related other inflammatory disease of the selected microRNA candidates. [file 12967_2018_1565_MOESM1_ESM.docx]

|  | Regulation status | Biomarker | Candidate gene | Involved signaling pathway | Related other inflammatory disease (except Graves’ disease) |
| --- | --- | --- | --- | --- | --- |
| miR-142-3p | Down-regulation or up-regulation | PBMCs, thyroid tissue, serum | BACH 1(BTB and CNC homology 1); BACH 2(BTB and CNC homology 2); FOXO4 (forkhead box O4), ITGAV (integrin, alpha V); TGFBR (transforming growth factor, beta receptor 1); et al. | Bacterial invasion of epithelial cells; B cell receptor signaling pathway; TNF signaling pathway; et al. | Dermatitis, Atopic; Inflammation; Psoriasis; Multiple sclerosis, Relapsing-Remitting; Chronic Periodontitis; Lupus Nephritis; Inflammatory Bowel disease; Epstein-Barr Virus; HIV infections |
| miR-154-3p | Down-regulation | PBMCs | ZEB2 (zinc finger E-box binding homeobox 2); USP34 (ubiquitin specific peptidase 34); SIRT1 (sirtuin 1); FGF9(fibroblast growth factor 9); et al. | adheres junction; PI3K-AKT; et al. | Pneumonia; Chronic disease; Muscular dystrophies |
| miR-431-3p | Down-regulation | PBMCs | ITGA11 (integrin, alpha 11); BCL9 (B cell CLL/lymphoma 9); FOXP1 (forkhead box P1); AGO4 (argonaute RISC catalytic component 4); et al. | ECM receptor interaction; et al. | Multiple sclerosis |
| miR-590-5p | Down-regulation | PBMCs | TIAM1 (T-cell lymphoma invasion and metastasis 1); TGFB1 (transforming growth factor, beta-induced); SAMD7; FGF18 (fibroblast growth factor 18); TNFRSF11B (tumor necrosis factor receptor superfamily, member11b); FOXP3 (forkhead box P3); et al. | Endocrine and other factor-regulated calcium reabsorption; TGF-β signaling pathway; et al. | Diabetes mellitus |
| let-7b | Up-regulation | PBMCs | ZBTB5 (zinc finger and BTB domain containing 5), ZBTB16 (zinc finger and BTB domain containing 16); FOXP2 (forkhead box P2); IGF1R (insulin-like growth factor 1 receptor); TGFBR1 (transforming growth factor, beta receptor 1); et al. | p53 signaling pathway; Bacterial invasion of epithelial cells; TGF-β signaling pathway; thyroid hormone pathway; et al. | Cytomegalovirus Infections; Lymphoproliferative Disorders; Epstein-Barr Infections; Sepsis; Rheumatoid Arthritis; Tuberculosis; Helicobacter Infections; Ascites; Chronic disease; Inflammation; Diabetes mellitus |

Additional file 1: Table S1. The predicted candidate gene, pathway and related other inflammatory disease of the selected microRNA candidates
